# Management of Reduced Bone Mineral Density in HIV: Pharmacological Challenges and the Role of Exercise

**DOI:** 10.3389/fphys.2018.01074

**Published:** 2018-08-07

**Authors:** Enock M. Chisati, Demitri Constantinou, Fanuel Lampiao

**Affiliations:** ^1^Department of Physiotherapy, College of Medicine, University of Malawi, Blantyre, Malawi; ^2^Center for Exercise Science and Sports Medicine, University of the Witwatersrand, Johannesburg, South Africa; ^3^Physiology Unit, Department of Biomedical Sciences, College of Medicine, Blantyre, Malawi

**Keywords:** bone mineral density (BMD), antiretroviral therapy (ART), people living with HIV (PLWHIV), exercise, progressive resistance exercise (PRE), osteopenia, osteoporosis

## Abstract

Low bone mineral density is becoming more common among people living with HIV following the use of current antiretroviral therapy drugs such as tenofovir. Although pharmacological therapies used to treat low bone mineral density are associated with adverse effects and may increase the pill burden in people living with HIV who are already burdened by antiretroviral therapy drugs, non-pharmacological strategies to prevent and treat reduced bone mineral density resulting from antiretroviral therapy drugs in people living with HIV have not been fully explored. Despite evidence that exercise is effective in increasing bone mineral density, effects of exercise on low bone mineral density resulting from antiretroviral therapy drugs in HIV infected individuals are still unknown. This review highlights gaps in the strategies used to manage reduced bone mineral density resulting from antiretroviral therapy drugs and focuses on exercise as an alternative or adjunctive strategy.

## Introduction

Despite benefits of increased survival, use of the current antiretroviral therapy (ART) drugs in HIV patients are associated with reduced bone mineral density(BMD) (Purdy et al., 2008; Haskelberg et al., 2012; Alonge et al., 2013; Dave et al., 2015; Escota et al., 2015; Chitu-Tisu et al., 2016; Matovu et al., 2016; Mirembe et al., 2016). Reduced BMD is characterized by osteopaenia and osteoporosis which predispose people living with HIV(PLWHIV) to future fall related fractures thereby increasing the risk for morbidity and mortality. The prevalence of osteoporosis and osteopenia in people living with HIV and receiving ART is estimated to be over three times higher than that in HIV uninfected individuals (Brown and Qaqish, 2006). However, despite the widespread use of ART drugs among people living with HIV (World Health Organisation, 2015a), there is no consensus on effective strategies to manage reduced bone mineral density resulting from ART (Matovu et al., 2016).

Effective strategies to prevent and treat reduced bone mineral density in PLWHIV and receiving ART have no clear directions. Pharmacological strategies to manage reduced BMD resulting from ART include medications such as bisphosphonates, teriparatide and denosumab as well as providing vitamin D and calcium supplements (Ali et al., 2014). However these pharmacological therapies (Gallagher and Sai, 2010; Harris and Brown, 2012; Ali et al., 2014) are associated with adverse effects such as tumors, infection, nasopharyngitis, osteosarcoma as well as bronchitis (Kendler et al., 2010; Harris and Brown, 2012; Ali et al., 2014) which limit their recommendation for use in HIV infected individuals (Harris and Brown, 2012). In addition, the current cost of treating bone loss using pharmacological therapies such as bisphosphonates is prohibitive (Matovu et al., 2016). Further, pharmacological therapies may increase the pill burden in people living with HIV who are already burdened by ART. Physical activities such as jogging, walking, dancing and weight lifting are also recommended as non-pharmacological strategies for preventing and treating bone loss (Howe et al., 2011), but the effectiveness of physical activity in increasing bone mass resulting from ART in people living with HIV has not been fully elucidated.

Although there is growing evidence that physical activity and exercise increases bone mineral density in both adult men and women (Ryan et al., 2004; Cheung and Giangregorio, 2012; Mosti et al., 2014; Multanen et al., 2015), the effects of exercise on loss of bone mass resulting from antiretroviral drugs in PLWHIV remains unexplored (Grace et al., 2015). Exercise programmes differ in terms of frequency, intensity, duration and type. Among many studies, there is heterogeneity in the type, intensity, frequency and duration of exercise interventions to increase bone mineral density (Bolam et al., 2013) with most trials conducted in either women or adult men (Howe et al., 2011; Mosti et al., 2013, 2014) despite evidence of increases in bone loss among young men as well (Watts et al., 2012). Although reduced bone mineral density is common among PLWHIV following the use of antiretroviral drugs (Purdy et al., 2008; Haskelberg et al., 2012; Alonge et al., 2013; Dave et al., 2015; Escota et al., 2015; Chitu-Tisu et al., 2016; Matovu et al., 2016; Mirembe et al., 2016) knowledge on effects of exercises in increasing bone mineral density in this patient group is still lacking.

This review will highlight knowledge gaps in strategies to manage bone loss resulting from ART. The review will focus on challenges of pharmacological strategies used in treating reduced bone mineral density resulting from ART and the effects of exercises in increasing bone mineral density.

## Methods

From April to September 2017, online databases such as EMBASE, Google Scholar, MEDLINE, PubMed, Scopus and The Cochrane Library were searched with no period restriction using key words: bone mineral density, antiretroviral therapy, exercise, people living with HIV and progressive resistance exercise. Published articles with potentially relevant titles and abstracts were retrieved. Articles were included in the review if they were (i) investigating the prevalence of low bone mineral density resulting from ART; (ii) examining strategies that are used to treat reduced bone mineral density or (iii) investigating the effects of exercise in increasing bone mineral density. A total of 109 articles met the inclusion criteria and were included in the review. All included publications were reviewed in their entirety.

## Physiology of bone mineral density

Bone is a connective tissue in which the matrix is made up of collagen fibers and minerals. Collagen is a protein that provides the bone's flexible framework (Sharp, 2011). The minerals contribute to the bone mineral density or bone mineral content and give the bone its strength and hardness. Collagen allows bones to bend in order to withstand stress while bone mineral density give bones strength to support the body's other tissues (Sharp, 2011). Bone mineral density in the matrix contributes to the support and protection functions of the skeleton. As such, bone mineral density is a surrogate for bone strength and is used to predict fracture risk in an individual. Reduced bone mineral density is characterized by osteopenia and osteoporosis and can predispose an individual to future fall related fractures.

Bone grows through modeling and remodeling processes (Creager, 1992; Harada and Rodan, 2003; Seeman and Delmas, 2006; Marieb and Hoehn, 2007; Pavy-Le Traon et al., 2007; Guadalupe-Grau et al., 2009; Sharp, 2011; Kruger and Nell, 2017). The modeling process involves osteoblast cells that perform bone formation by laying down new bone (Sharp, 2011). On the other hand, remodeling is a continuous process which involves bone resorption. In resorption osteoclasts are attracted to areas needing repair and move in to remove damaged bone (Seeman and Delmas, 2006; Sharp, 2011) thus some bone tissue is added along one surface while reabsorption occurs at another surface. The remodeling process occurs throughout one's life. It is estimated that 5–7% of bone mass is remodeled every week and approximately 0.5 gram of calcium is deposited or reabsorbed by the adult skeleton daily (Marieb and Hoehn, 2007). Any imbalances between modeling and remodeling lead to reduced load bearing capacity as well as loss of bone mineral density which in turn increase the risk for fractures (Sharp, 2011). Therefore, increased bone mineral density may reduce the incidence of fractures.

**Figure 1:**
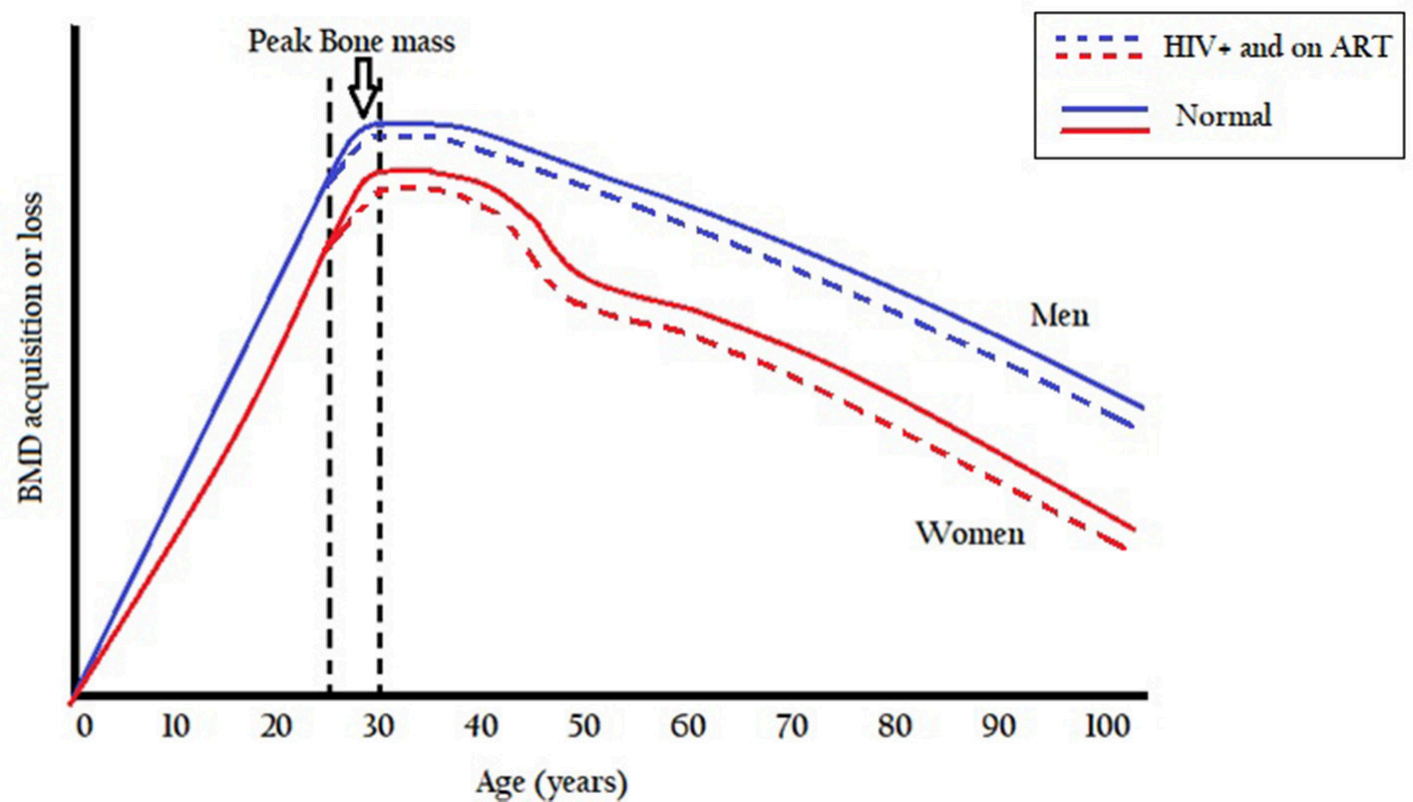
Bone mass growth.

Bone mass formation is normally in excess of reabsorption with increasing age and peaks between ages 25 and 30 years, and thereafter bone mass starts to decrease leading to lower bone mineral density (Seeman and Delmas, 2006). Modeling and remodeling processes during growth are aimed at establishing peak bone mass so as to maintain bone strength in adulthood (Seeman and Delmas, 2006). During childhood growth spurt, bone mineral density accumulates with the bone growing both in size and strength (Kruger and Nell, 2017). After the growth spurt, usually during the pre- and post-adolescent period, bone formation continues until a peak bone mass is reached between ages 25 and 30 years. The age of attainment of peak bone mineral density is site-specific with gains of about 5–12% in bone mineral density observed after 30 years old in other individuals (Lorentzon et al., 2005). After the third decade, bone mineral density is maintained for about 10 years before it starts to decline at a rate of about 0.3–0.5% per year in both males and females (Baxter-Jones et al., 2011; Kruger and Nell, 2017). At ages between 45 and 55 women lose more bone mineral than men after which the rate of bone loss is gradual and the same in both sexes (Figure 1). A rapid loss of bone mineral density in women between ages 45 and 55 years is possibly due to a decrease in estrogen production as the menstrual cycle ceases during this period (Kruger and Nell, 2017).

Although bone remodeling is a normal and natural process, some factors are thought to disrupt the remodeling process (Kruger and Nell, 2017) and thereby reduce or increase the rate of bone mineral density. Factors such as; physical inactivity, low body weight, nutritional deficiencies especially of calcium and vitamin D, depression, smoking, heavy alcohol use, corticoids and other medications, including more recently some antiretroviral drugs (Purdy et al., 2008; Harris and Brown, 2012; Haskelberg et al., 2012; Alonge et al., 2013; Ameet et al., 2014; Dave et al., 2015; Escota et al., 2015; Chitu-Tisu et al., 2016; Matovu et al., 2016; Mirembe et al., 2016) are associated with reduced bone mineral density. Although some factors are not modifiable, physical activity can be changed to stimulate greater accumulation of peak bone mass (Michelson et al., 1996; Guadalupe-Grau et al., 2009; Welz et al., 2010; Pinto Neto et al., 2011; Mdodo et al., 2015; Kruger and Nell, 2017). Since bone modeling and remodeling depends, in part, on mechanical stress, bone strength is enhanced or reduced in response to increased or reduced mechanical loading. Hence an individual's physical activity lifestyle can play a role in either increasing or reducing bone mineral density.

## Bone mineral density and art

Despite benefits of increased survival, anti-retroviral therapy has been shown to significantly contribute to loss of bone mass (Purdy et al., 2008; Harris and Brown, 2012; Haskelberg et al., 2012; Alonge et al., 2013; Ameet et al., 2014; Dave et al., 2015; Escota et al., 2015; Chitu-Tisu et al., 2016; Matovu et al., 2016; Mirembe et al., 2016). Consequently, medical comorbidities such as osteoporosis and fragility fractures resulting from low bone mineral density are on the rise (Battalora et al., 2014). Emerging evidence indicates that HIV infection is strongly associated with a fivefold increased risk for hip fractures independent of age, gender and comorbidities (Güerri-Fernandez et al., 2013). Güerri-Fernandez et al. reported an increased risk for hip fractures (hazard ratio, 6.2) among HIV infected patients compared to a non-HIV infected general population (Güerri-Fernandez et al., 2013). This risk is higher compared to the risk of lung cancer (hazard ratio, 3.6) and a combined risk of cardiovascular and pulmonary diseases (odds ratio, 1.58) among people living with HIV (Kirk et al., 2007; Schouten et al., 2014). Therefore, the increased risk for hip fractures could consequently increase the risk for mortality and morbidity in people living with HIV.

Initiation of antiretroviral therapy has been shown to increase bone loss in people living with HIV irrespective of regimen (Yin and Overton, 2011; Grant and Cotter, 2016). Reports show a decrease of about 2–6% in bone mineral density in the first two years of ART initiation regardless of the regimen (Duvivier et al., 2009). Although mechanisms underlying bone loss resulting from ART are unclear, it has been suggested by Duvivier (Duvivier et al., 2009) and Borderi (Borderi and Pierluigi, 2013) that HIV infection of osteoblasts may be related to a negative balance of bone remodeling thereby leading to a reduction in bone mineral density in people living with HIV (Duvivier et al., 2009; Borderi and Pierluigi, 2013). Despite suggestions that the likelihood of HIV infection of osteoblasts is very low due to low expression of CD4 (Nachera et al., 2001; Borderi et al., 2009), recent evidence suggests that higher levels of C-C chemokine receptor 5 (CCR5) may affect the functional regulation of osteoclasts thereby leading to bone loss (Lee et al., 2017).

While decreases in bone mineral density occur at initiation of ART irrespective of regimen, more bone loss is associated with tenofovir-containing regimens than other regimens (McComsey et al., 2011; Brown et al., 2015; Grant and Cotter, 2016; Matovu et al., 2016). Tenofovir leads to approximately 1–3% greater bone mineral loss compared to non tenofovir-containing regimens (Grant and Cotter, 2016). McComsey et al. (2011) compared the effects of tenofovir vs. other ART regimens on bone mass in PLWHIV. They observed greater decreases in spine and hip BMD in participants treated with tenofovir than those treated with other regimens (McComsey et al., 2011). This could suggest that tenofovir has an independent effect on bone regardless of host, viral and immunological factors. Despite evidence that tenofovir significantly contributes to loss of bone mass (McComsey et al., 2011; Brown et al., 2015; Grant and Cotter, 2016; Matovu et al., 2016), most first line ART treatment regimens recommended by World Health Organization (WHO) in resource-limited settings, contain tenofovir (World Health Organisation, 2013, 2015b). This makes reduced bone mineral density highly likely among people living with HIV in most resource-limited settings yet strategies to minimize bone loss in PLWHIV in these settings are currently lacking (Matovu et al., 2016).

Although ART contributes to bone loss in people living with HIV, other factors may play a role as well. Traditional factors such as physical activity, lower body mass index, female sex, older age, nutritional deficiencies of calcium and vitamin D, depression, smoking and alcohol use are believed to contribute to loss of bone mass in the general population (Michelson et al., 1996; Guadalupe-Grau et al., 2009; Welz et al., 2010; Pinto Neto et al., 2011; Mdodo et al., 2015). While there is a controversy on traditional risk factors contributing to reduced bone mineral density in PLWHIV(Michelson et al., 1996), other authors have demonstrated that risk factors for reduced bone mineral density in HIV are similar to other populations (Bonjoch et al., 2010). Other reports indicate that poverty may also contribute to low bone mineral density (Navarro et al., 2009) suggesting that PLWHIV in resource-limited settings could be at a higher risk for loss of bone mineral mass, often the setting of highest HIV burden.

Evidence is emerging that HIV severity also contributes to reduced bone mineral density in HIV infected individuals receiving ART (Ofotokun et al., 2012, 2016; Battalora et al., 2014). A study by Grant et al. demonstrated that with ART initiation, HIV infected individuals with a low CD4 cell count (<50 cells/mm^3^) had greater bone loss than those with a higher CD4 cell count (>500 cells/mm^3^) (Grant et al., 2013). This indicates that chronic inflammation induced by HIV may impact bone metabolism. However, despite regional increases in HIV inflammation (Kaul et al., 2011, 2015), data on effective strategies to manage bone loss in people living with HIV lack clear guidelines.

## Management of bone loss resulting from art

### Pharmacological therapies

Treatment strategies for reduced bone mineral density resulting from anti-retroviral therapy have no clear directions. Pharmacological strategies to manage bone loss resulting from antiretroviral therapy include providing vitamin D and calcium supplements as well as pharmacological therapies such as Bisphosphonates, Teriparatide and Denosumab (Ali et al., 2014). This section will discuss limitations of vitamin D and calcium supplements as well as pharmacological therapies in treating reduced bone mineral density in people living with HIV.

Although vitamin D deficiency has been implicated in the pathogenesis of bone loss in people living with HIV (Harris and Brown, 2012), there are no clear recommendations for vitamin D and calcium supplements to treat low bone mineral density in this population (Lima et al., 2011). A study by Dao reported a 70.3% vitamin D deficiency in a cohort of 672 HIV infected participants (Dao et al., 2011). Factors such as African American race and exposure to ART drugs were found to be associated with increased risk to vitamin D deficiency (Dao et al., 2011). This could suggest that vitamin D deficiencies could be higher among the Afro American race, moreover it is in Africa where the use of ART is becoming common as a result of high prevalence rates of HIV. A review by Harris and Brown (2012) recommends higher doses of vitamin D in people living with HIV exhibiting bone loss to maintain targeted levels of bone mass (Harris and Brown, 2012). However, due to their small effect on fracture risk reduction, vitamin D and calcium supplements are best used as additional therapies with other osteoporotic drugs (Gallagher and Sai, 2010) and their sole use is not advised.

Among osteoporotic drug treatments, beneficial effects of Denosumab in managing reduced bone mineral density in people living with HIV are not clear (Ali et al., 2014). Denosumab is a long acting monoclonal antibody that blocks bone resorption (Ali et al., 2014). Denosumab decreases osteoclastogenesis and is recommended for use in persons with a history of osteoporotic fractures or those who are intolerant to other osteoporotic therapies (Harris and Brown, 2012). However, long term use of denosumab in treating bone loss leads to atypical fractures (Sellmeyer, 2010). Although seemingly effective, use of Denosumab brings adverse effects such as, tumors, infection, nasopharyngitis, back pain, bronchitis and arthralgia (Kendler et al., 2010; Harris and Brown, 2012). These adverse effects are of particular concern to people living with HIV considering that they are already at an increased risk for infection.

Although available in resource limited settings and could be an alternative to denosumab for treating bone loss, bisphosphonates have a number of side effects which are a cause of concern in people living with HIV. Bisphosphonates, available as alendronate, ibandronate, risedronate, and zoledronic acid are said to decrease fracture risks in some parts of the body by between 25 and 50% in the general populations (Dennis et al., 1996; Ettinger et al., 1999; Harris et al., 1999; Black et al., 2007). However, despite improvements in bone mineral density among HIV infected individuals following use of alendronate and zoledronic acid, side effects such as difficulty swallowing, esophageal inflammation, dyspepsia, and gastric ulcer are also observed (Harris and Brown, 2012). In addition, bisphosphonates induce atypical femoral fractures and may not be used for more than 5 years (Fleming et al., 2001; Ali et al., 2014). This raises concerns of the long term effects of using bisphosphonates for managing reduced bone mineral density in HIV infected individuals who are currently living longer as a result of ART.

While teriparatide is recommended in individuals where bisphosphates have failed, its recommendation for use to treat bone loss in people living with HIV is still controversial. Some reports indicate that teriparatide has a risk for osteosarcoma (Ali et al., 2014), albeit rare, which may limit its recommendation for use in HIV infected individuals. Additionally, a review by Harris and Brown (2012) concluded that data on safety and efficacy of teriparatide in people living with HIV is lacking and requires further investigation (Harris and Brown, 2012).

Apart from the many challenges associated with pharmacological therapies in treating bone loss in HIV infected individuals, compliance and adherence issues have also been associated with pharmacological therapies (Brown, 2013). A retrospective study by Fan et al. (2013) which assessed the level of compliance with drugs prescribed for bone loss for seven years concluded that most patients do not continue to take the medication as prescribed (Fan et al., 2013). It has also been observed that half of patients treated with bisphosphates discontinue with treatment after 4 months (Solomon et al., 2005; Fan et al., 2013). Since pharmacological therapies are associated with a number of side effects and adherence problems which may limit their use among HIV infected individuals on ART (Fleming et al., 2001; Solomon et al., 2005; Kendler et al., 2010; Sellmeyer, 2010; Harris and Brown, 2012; Brown, 2013; Fan et al., 2013; Ali et al., 2014), exercise based interventions could be an attractive alternative.

### Physical activity

Guidelines for good bone health include physical activity and exercise as a major component in preventing bone loss (Body et al., 2011; Sharp, 2011; Borderi and Pierluigi, 2013; Cosman et al., 2014). Physical activity has been suggested as a non-pharmacological strategy that can be used to increase bone mineral density even in people living with HIV (Sharp, 2011; Borderi and Pierluigi, 2013). Among others, physical activities such as jogging, walking, dancing and weight lifting are shown to be beneficial in preventing and treating low bone mineral density (Howe et al., 2011). However, evidence that physical activity is related to higher bone mass is often inappropriately interpreted as evidence that any activity will improve bone mass (Beck et al., 2016).

Contrary to reports that all physical activity could be important in increasing bone mineral density (Body et al., 2011), weight bearing physical activities with high force, yield a notable increase in bone mineral density (Howe et al., 2011). This could suggest that the type and intensity of the physical activity has an additive effect on bone density. Although weight bearing physical activities are recommended to improve BMD, appropriate parameters for frequency, intensity, duration and type of physical activity to increase bone mineral density especially among HIV infected individuals has not been fully explored (Schambelan et al., 2002; Cosman et al., 2014).

## Exercise and bone mineral density

### Type and design of exercises

There is growing evidence that exercise increases bone mineral density (Ryan et al., 2004; Cheung and Giangregorio, 2012; Mosti et al., 2014; Multanen et al., 2015). However, not all types of exercises provide notable stimulus to bone (Guadalupe-Grau et al., 2009; Xu et al., 2016). Aerobic exercises such as swimming, walking and cycling provide insignificant improvement to bone mineral density (Martyn-St James and Carroll, 2008; Rector et al., 2008; Ma et al., 2013). Simply prescribing these exercises in isolation is insufficient to optimize bone health. Bone respond positively to impact activities and high intensity progressive resistance training (Beck et al., 2016; Bolam et al., 2016). For example, a Cochrane review (Howe et al., 2011) on the effects of exercise on bone mineral density in postmenopausal women reported that exercises such as jumping, jogging, or dancing results in a between group difference in favor of exercise at the hip (1.55%) but not at the lumbar spine (−1.22%). Similarly, exercises such as walking showed between group improvement with exercise at the lumbar spine (0.85%) but not at the femoral neck (−1.20%) (Howe et al., 2011). Yet progressive resistance exercises resulted in significant between group differences in favor of exercise at both the femoral neck (1.03%) and lumbar spine (0.86%) (Howe et al., 2011). Results from this review suggest that progressive resistance exercises may be effective in increasing bone mineral density. However, despite reports of increases in bone loss due to ART (Yin and Overton, 2011; Grant and Cotter, 2016), the impact of progressive resistance exercise in increasing bone mineral density in PLWHIV has not been fully investigated nor promoted.

Although progressive resistance exercises have been shown to increase bone mass in the general population (Fairfield et al., 2001; Ryan et al., 2004; Ahola et al., 2009; Bailey and Brooke-Wavell, 2010; Ciccolo et al., 2010; Morseth et al., 2010; Howe et al., 2011; Kukuljan et al., 2011; Marques et al., 2011; Cheung and Giangregorio, 2012; Langsetmo et al., 2012; Watts et al., 2012; Allison et al., 2013; Kelley et al., 2013a,b; Mosti et al., 2013, 2014; Behringer et al., 2014; Kemmler and von Stengel, 2014; Hinton et al., 2015; Hui et al., 2015; Multanen et al., 2015; Kemmler et al., 2016), there is heterogeneity in the type, intensity, frequency and duration of exercise interventions to increase bone mineral density among many studies (Bolam et al., 2013). In a longitudinal randomized trial, Allison et al. (2013) investigated the influence of 12 months high impact exercises on bone mineral density in 50 men. Results of the study revealed an increase of 1.2% in bone mineral density. Similarly, Bailey and Brooke-Wavell (2010) demonstrated a significant increase in BMD after 6 months of exercise in 65 women compared to 20 non-exercising women. However, in both studies, there was an increase in dropout rates with increasing number of exercise days indicating that long exercise durations could lead to exercise adherence problems.

Adherence to the recommended exercise regimen is key to the success of any exercise intervention. The World Health Organization defines adherence as “the extent to which a person's behavior—taking medication, following a diet, and/or executing lifestyle changes—corresponds with agreed recommendations from health care provider” (World Health Organisation, 2003). In exercise, adherence refers to complying with an exercise design for a specified period of time. It involves maintaining the frequency, intensity, duration and type of a given or prescribed exercise. There are reports that adherence to exercise falls below the desirable level among people living with HIV (Petróczi et al., 2010). However, reports indicate that exercise interventions yield higher adherence rates compared to pharmacological interventions in treating low bone mineral density (Kelley and Kelley, 2013).

Among the different types of exercise interventions, compliance is higher with progressive resistance exercises than aerobic exercises(Vancampfort et al., 2017) with an adherence rate of over 80% in randomized controlled trials (Aitken et al., 2015). Reports also indicate that facility based exercises with shorter durations (Kelley and Kelley, 2013) such as maximal strength exercises (Mosti et al., 2013, 2014) have increased adherence. In addition, adherence is increased in exercise programmes that are individualized and supervised by qualified professionals (Hong et al., 2008; Jordan et al., 2010; Tønnesen et al., 2016). It is important therefore to design shorter, supervised, individualized and facility based progressive resistant exercise programmes targeting BMD in order to increase adherence.

### Progressive resistance exercises

Progressive resistance exercises have proven to be beneficial among people living with HIV. A systematic review by O'Brien et al, found no significant differences in CD4 count and viral load in HIV infected individuals before and after participating in progressive resistance exercises for at least three times per week for at least 6 weeks demonstrating that exercises are safe for people living with HIV (O'Brien et al., 2017). Significant improvements in cardiorespiratory fitness, strength, body composition and body weight following participation in progressive resistance exercises have also been reported among people living with HIV (O'Brien et al., 2008, 2017; Neto et al., 2015). Although the benefits of progressive resistance exercise in people living with HIV are wide, effects of such exercises on bone mineral density in this population have not been fully evaluated (Grace et al., 2015). Only one study by Santos et al. (2015) investigating effects of progressive resistance exercise on BMD in PLWHIV was identified from the literature. In this study, Santos et al. (2015), demonstrated that a shorter exercise duration of 12 weeks was appropriate to impact significant bone increases in 20 individuals living with HIV.

However, the study by Santos et al. (2015) has some methodological shortcomings. The exercise design used lacked other basic elements of an appropriate exercise programme to elicit improvements (Slade and Keating, 2011). The trial neither used non HIV infected controls nor local reference data for BMD for comparison. In addition, different types of exercises were used raising concerns of which of the exercises had a greater impact on bone mineral density. The effectiveness of a 12 weeks' exercise duration to improve BMD is supported by evidence from studies by Mosti et al. (2013, 2014), who demonstrated the effects of a 12 weeks progressive resistance exercises in increasing BMD in young women and postmenopausal women (Mosti et al., 2013, 2014). This evidence indicate that shorter exercise durations could as well impact bone metabolism, thereby minimizing exercise adherence problems.

Progressive resistance exercises have been proven to be safe and beneficial in improving metabolic outcomes among PLWHIV (O'Brien et al., 2008; Fillipas et al., 2010; Grace et al., 2015; Neto et al., 2015). However, there is still lack of knowledge on the optimal mode of frequency, duration and intensity of progressive resistance exercise on BMD in people living with HIV (Fillipas et al., 2010; Grace, 2016) which requires investigation.

## Conclusion

Most pharmacological strategies used to treat bone loss are associated with a number of adverse effects which limit their recommendation for use in PLWHIV. In addition, compliance and adherence issues associated with pharmacological strategies in treating bone loss may limit their use among PLWHIV who are already burdened by ART. Exercise based interventions such as progressive resistance exercises seem to be an attractive safe and effective alternative strategy that could be used to manage bone loss resulting from ART in PLWHIV. Although progressive resistance exercises are effective in increasing BMD, there is lack of knowledge on the optimal frequency, intensity and duration of the exercise to impact bone which need further investigations. In addition, effects of progressive resistance exercises in increasing BMD in PLWHIV have not been fully investigated. Only one study examining the effects of progressive resistance exercise in increasing BMD among PLWHIV and receiving ART was identified from the literature. Future studies investigating the effects of progressive resistance exercises in increasing BMD in PLWHIV should adopt trial designs with clear descriptions of exercise frequency, intensity and duration.

## Author contributions

EC, DC, and FL made significant contributions to the conception and design of the work. EC drafted the work. DC and FL critically revised the work for important intellectual content. All authors had final approval of the version to be published, and are in agreement to be accountable for all aspects of the work in ensuring that questions related to the accuracy or integrity of any part of the work are appropriately investigated and resolved.

### Conflict of interest statement

The authors declare that the research was conducted in the absence of any commercial or financial relationships that could be construed as a potential conflict of interest.
